# Tumor morphology and location associate with immune cell composition in pleomorphic sarcoma

**DOI:** 10.1007/s00262-021-02935-2

**Published:** 2021-04-17

**Authors:** Rosanna L. Wustrack, Evans Shao, Joey Sheridan, Melissa Zimel, Soo-Jin Cho, Andrew E. Horvai, Diamond Luong, Serena S. Kwek, Lawrence Fong, Ross A. Okimoto

**Affiliations:** 1 Department of Orthopedic Surgery, University of California, San Francisco, USA; 2 Division of Hematology and Oncology, Department of Medicine, University of California, 513 Parnassus Avenue, HSW1201, San Francisco, CA 94143 USA; 3 Department of Pathology, University of California, San Francisco, USA; 4 Helen Diller Comprehensive Cancer Center, University of California, San Francisco, USA; 5 Parker Institute of Cancer Immunotherapy, University of California, San Francisco, USA

**Keywords:** Immune-based profiling, Pleomorphic sarcoma, Soft-tissue sarcoma

## Abstract

**Background:**

Soft-tissue sarcomas (STS) are a rare group of mesenchymal malignancies that account for approximately 1% of adult human cancer. Undifferentiated pleomorphic sarcoma (UPS) is one of the most common subtypes of adult STS. Clinical stratification of UPS patients has not evolved for decades and continues to rely on tumor-centric metrics including tumor size and depth. Our understanding of how the tumor microenvironment correlates to these clinicopathologic parameters remains limited.

**Methods:**

Here, we performed single-cell flow cytometric immune-based profiling of 15 freshly resected UPS tumors and integrated this analysis with clinical, histopathologic, and outcomes data using both a prospective and retrospective cohort of UPS patients.

**Results:**

We uncovered a correlation between physiologic and anatomic properties of UPS tumors and the composition of immune cells in the tumor microenvironment. Specifically, we identified an inverse correlation between tumor-infiltrating CD8 + T cells and UPS tumor size; and a positive correlation between tumor-infiltrating CD8 + T cells and overall survival. Moreover, we demonstrate an association between anatomical location (deep or superficial) and frequency of CD4 + PD1^hi^ infiltrating T cells in UPS tumors.

**Conclusions:**

Our study provides an immune-based analysis of the tumor microenvironment in UPS patients and describes the different composition of tumor infiltrating lymphocytes based on size and tumor depth.

**Supplementary Information:**

The online version contains supplementary material available at 10.1007/s00262-021-02935-2.

## Introduction

Soft-tissue sarcomas (STS) are a heterogenous group of mesenchymal tumors characterized by immense histological, molecular, and clinical diversity [1, 2]. The rarity and diverse histological complexity of STSs poses a unique challenge in identifying universal predictive and prognostic biomarkers across all sarcoma subtypes [3]. Several staging systems have been employed over the years that attempt to more accurately prognosticate STS patients, which frequently rely on histological grade, tumor size, and tumor depth [4, 5]. While these tumor-centric parameters have provided clinical value for decades, our understanding of how these clinicopathologic features impact tumor immune cell infiltration remains limited.

The tumor microenvironment plays a key role in sarcoma progression and therapeutic response [6–9]. However, whether tumor physiology and anatomic location influence the host immune response remains largely unknown. Thus, understanding the relationship between tumor growth (physiologic) and tumor depth (anatomic) and the immunologic response can enhance our understanding of STS progression and potentially identify immune-cell subsets that aid in the therapeutic stratification of STS patients.

In order to identify how tumor size and depth influences tumor immune cell infiltration, we developed a systematic platform to prospectively characterize the tumor-immune microenvironment of a single STS subtype, undifferentiated pleomorphic sarcoma (UPS). UPS is one the most common adult STS subtypes and is associated with responses to immune checkpoint inhibitors [10–13]. Furthermore, focused analysis on UPS tumors limits inter-tumoral heterogeneity and enables direct histological and clinical comparisons. Leveraging single-cell multiparameter flow cytometry, we systematically assessed the immune landscape of freshly resected UPS tumors derived from patients with clinical outcomes data. We then assessed the impact of specific host immune responses correlated with 5-year overall survival using a larger, retrospective cohort of UPS patients. Through the analysis of freshly resected UPS tumors, we reveal how the physiological properties and anatomical location of UPS tumors can potentially influence the host immune response.

## Results

### Single-cell assessment of undifferentiated pleomorphic sarcomas (UPS)

**Fig. 1:**
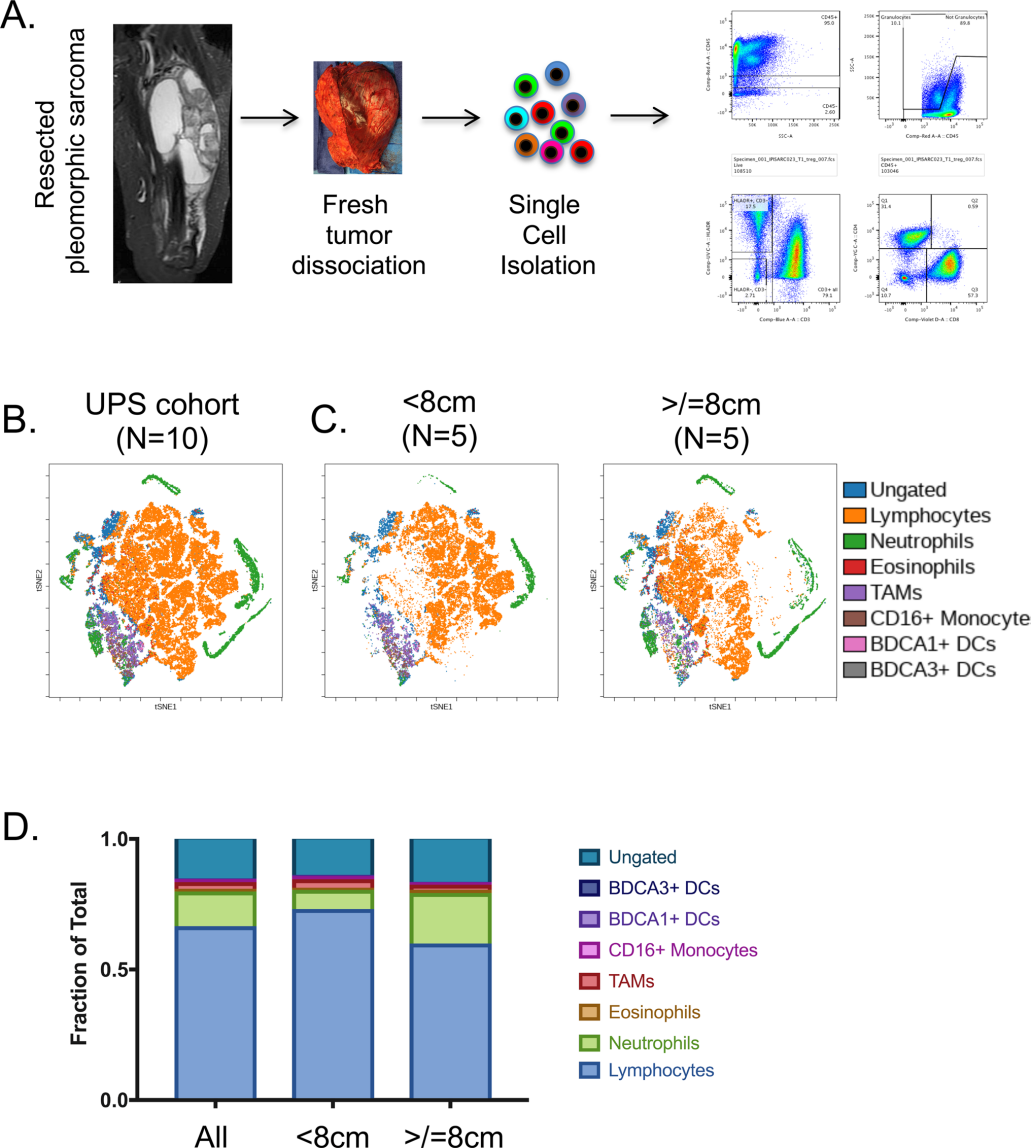
Single-cell immunoprofiling of the freshly resected UPS tumors. **a** Systematic approach to collect and profile the tumor-immune microenvironment of freshly resected pleomorphic sarcomas. **b** t-SNE plot analysis demonstrating immune cell populations in the entire UPS cohort. **c** t-SNE plot analysis comparing immune cell populations in UPS tumors less than or greater than 8 cm. **d** Stacked bar graph demonstrating the relative fraction of immune cell subsets in all UPS tumors, UPS tumors less than 8 cm, and UPS tumors greater than 8 cm

In order to map the UPS tumor immune microenvironment, we performed single-cell multi-parameter flow cytometry (MpFC) using 28 unique immune-cell specific markers across two panels (Fig. 1a). Our UPS cohort (Supplementary Tables 1–3) was established through systematic and sequential collection of UPS tumors. Nineteen freshly resected tumors were obtained from patients with biopsy-proven UPS of the extremities including hip girdle and buttock at time of wide resection (Table 1). Four patients’ specimens were removed from analysis due to the final pathology changing from UPS to a different sarcoma (N = 3) and lack of any viable cells for analysis in the sample (N = 1). Thus, the first 10 evaluable UPS tumors comprised a Discovery Cohort (N = 10) and subsequent collection of an additional five patients established the Expansion Cohort (N = 5) (Table 1). We leveraged the Discovery Cohort to generate an immune-cell portrait of the UPS tumor microenvironment (Fig. 1b). Through the Barnes–Hut acceleration of t-Distributed stochastic neighbor embedding (t-SNE) analysis, we captured high-dimensional data at single-cell resolution. Using this approach we first analyzed the distribution of distinct immune cell populations within UPS tumors collected in our Discovery Cohort (Fig. 1b and Supplementary Table 4). This analysis revealed the presence of all major immune cell lineages with the most abundant populations being lymphocytes and neutrophils (Fig. 1b). Tumor-associated macrophages (TAMs) were also found, but at low frequency. Collectively, these data enabled us to globally visualize the tumor immune microenvironment in freshly resected UPS tumors.

**Table 1:** Demographics, treatment, and clinical outcomes

	Entire UPS cohort	Discovery cohort	Expansion cohort
	N = 15	N = 10	N = 5
Follow-up months (range, SD)	20.2 (4–46, 12)	20.5 (4–46; 12.1)	19.6 (12–43; 13.1)
Lost to follow-up	0	0	0
Female	6 (40%)	4 (40%)	2 (40%)
Age (range, SD)	69.5 (48–85; 9.6)	69.3 (48–85;11.3)	70 (62–76; 6)
Stage			
II	4 (26.7%)	3 (30%)	1 (20%)
III	8 (53.3%)	4 (40%)	4 (80%)
IV	3 (20%)	3 (30%)	
Tumor size cm (range, SD)	9.8 (3–34; 8.1)	8.8 (3–21.5, 5.6)	11.7 (3–34; 12.5)
Deep	10 (66.7%)	6 (60%)	4 (80%)
Grade 3	14 (93.3%)	9 (90%)	5 (100%)
Received neoadjuvant chemotherapy	1 (6.7%)	1 (10%)	0 (0%)
Received neoadjuvant radiation	1 (6.7%)	1 (10%)	0 (0%)
Adjuvant radiation	13 (86.7%)	9 (90%)	4 (80%)
(IORT or EBRT)			
Local recurrence	1 (6.7%)	0	1 (20%)
Metastatic recurrence	3 (20%)	2 (20%)	1 (20%)
Current status			
NED	10 (66.7%)	8 (80%)	2 (40%)
AWD	2 (13.3%)	0	2 (40%)
DOD	2 (13.3%)	2 (20%)	0
DWD	1 (6.7%)	0	1 (20%)

### Clinicopathologic factors correlate with tumor infiltrating immune cell subsets

**Fig. 2:**
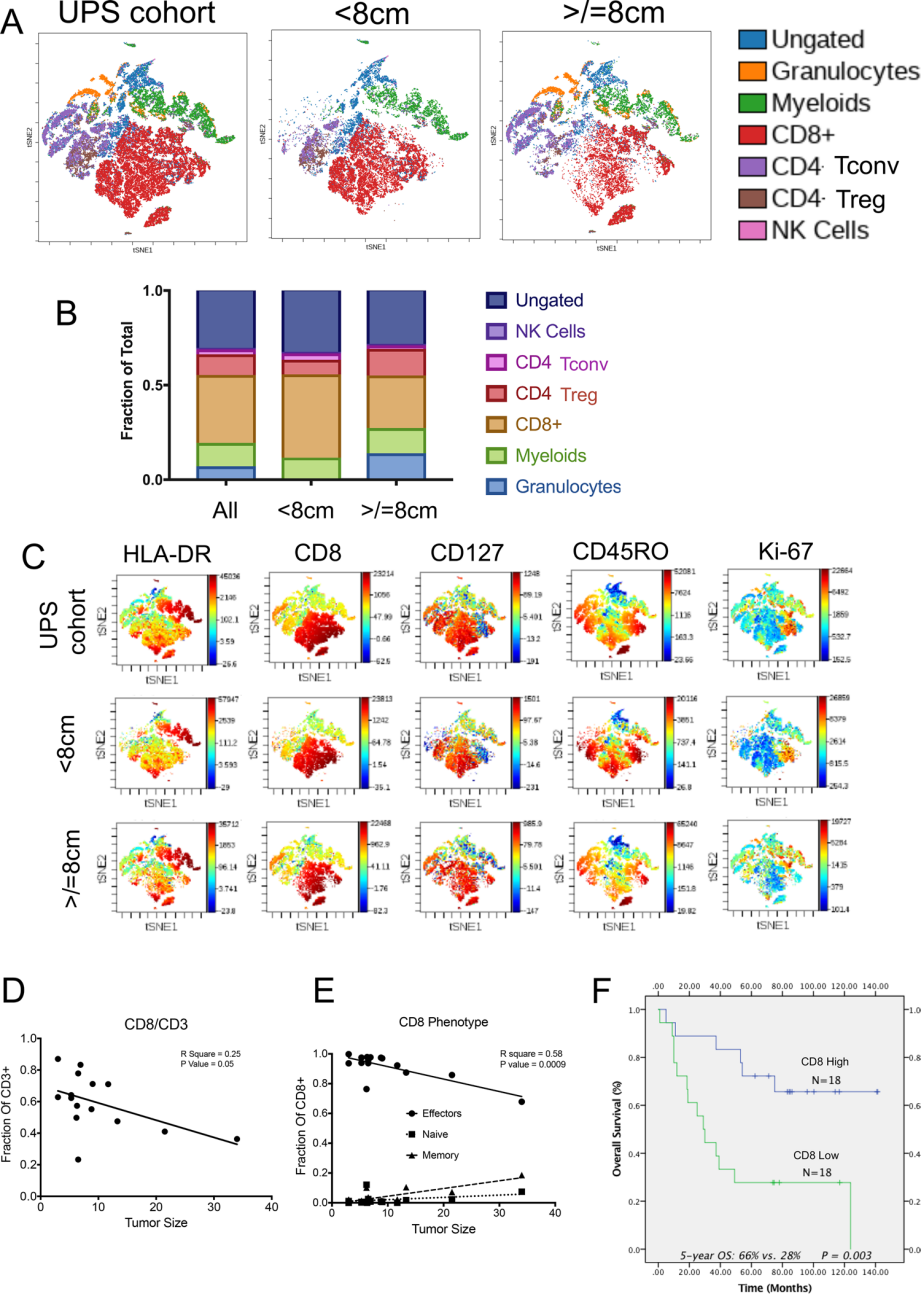
Impact of UPS tumor size on tumor infiltrating immune cells. **a** t-SNE plot analysis demonstrating T cell subsets, NK cells, myeloid, and granulocytes in the entire UPS cohort, UPS tumors < 8 cm, and UPS tumors > / = 8 cm. **b** Stacked bar graph depicting the relative fraction of immune cell subsets in the entire UPS cohort, UPS tumors < 8 cm, and UPS tumors > / = 8 cm. **c** Individual t-SNE channels demonstrating the expression of specific immune cell markers (HLA-DR, CD8, CD127, CD45RO, Ki-67). **d** Dot plot demonstrating the relationship between all CD8 + T cells and UPS tumor size. **e** Dot plot demonstrating the relationship between individual CD8 + T cell populations (effectors, naïve, and memory) and UPS tumor size. **f** Kaplan–Meier overall survival curve comparing UPS tumors with high CD8 + T cell expression to UPS tumors with low CD8 + lymphocyte expression. *p*-value, log-rank test

Clinicopathologic factors that impact survival in high-grade soft-tissue sarcoma (STS) remain controversial [3, 5]. However, tumor size and depth have been shown to correlate with clinical outcomes in patients with UPS [4]. Leveraging these established parameters, we wanted to identify how immune cell density evolved with tumor size and depth. To explore this we first used our Discovery Cohort to establish the mean tumor size (8 cm). We hypothesized that specific immune cell subsets would differentially infiltrate into the tumor microenvironment of tumors > / = 8 cm versus UPS tumors < 8 cm. Through our MpFC platform, we observed an increased frequency of neutrophils and a decreased frequency of total lymphocytes in UPS tumors > / = 8 cm compared to those < 8 cm (Fig. 1c–d). To further probe the specific identity of tumor-infiltrating lymphocytes (TILs), we performed a subset analysis using well-characterized immune-based markers to differentiate between CD3 + CD8 + (CD8), T-helpers CD3 + CD4 + CD25-FOXP3- (CD4 conv), CD3 + CD4 + CD25 + FOXP3 + (CD4 Tregs), CD56 + NK cells, myeloid and granulocytic populations (Supplementary Table 4). Through this analysis, we identified a decrease in the frequency of CD8 TILs in tumors > / = 8 cm compared to tumors < 8 cm (Fig. 2a–b). In order to establish the phenotype of these CD8 TILs, we first expanded our Discovery Cohort to include the Expansion Cohort and immuno-phenotypically clustered these CD8 cells into either effector (FOXP3-CD127-CD45RO ±), memory (FOXP3-CD127 + CD45RO +), or naive (FOXP3-CD127 + CD45RO-) states. Through this analysis, we observed a size-dependent decrease in the fraction of CD8 effector T cells (CD45 + /CD3e + /CD8 + /CD127-/CD25-) relative to CD8 + memory (CD45 + /CD3e + /CD8 + /CD127 + /CD25-/CD45RO +) or naïve cells (CD45 + /CD3e + /CD8 + /CD127 + /CD25-/CD45RO-) (Fig. 2c–e). Collectively, the decreased frequency of CD8 + effector T-cells in UPS tumors > / = 8 cm suggests that larger tumors may suppress immune surveillance to enhance tumor progression [6, 14–18].

Rationalizing that tumor size portends a poor prognosis in STS [4], we next examined whether CD8 + T-cell abundance also correlated with survival outcomes in patients with UPS tumors. Since our dataset was limited to fifteen patients, an overall survival analysis was not feasible at the time of publication. In order to address this, we leveraged a retrospective clinically annotated UPS dataset established at UCSF to assess the prognostic significance of CD8 + TILs in patients with UPS. Specifically, we retrospectively analyzed CD8 expression of 36 UPS tumors and found a significant correlation with low CD8 expression in TILs and worse overall survival (Fig. 2f). Collectively, these data indicate that as UPS tumors enlarge, CD8 + lymphocyte infiltration wanes, potentially compromising immune surveillance.

In a broader search to determine the relationship between tumor size and immune cell infiltration, we observed an increase in tumor infiltrating neutrophils in tumors > / = 8 cm compared to UPS tumors < 8 cm (Fig. 1c). Further analysis revealed that the neutrophil-to-lymphocyte ratio (NLR) increased in UPS tumors > / = 8 cm compared to tumors < 8 cm (NLR = 0.32 in tumors > / = 8 cm, 0.10 in tumors < 8 cm, *p*-value < 0.00001, Fisher’s-exact test) (Supplementary Table 5). Since increased systemic (blood-derived) NLR has been shown to be a strong prognostic indicator of poor clinical outcome in STS and other solid malignancies [19, 20], we theorize that elevated intratumoral NLR in UPS tumors may also predict worse disease outcome. This is consistent with recent observations in other solid tumors and warrants further investigation in sarcomas [21].

**Fig. 3:**
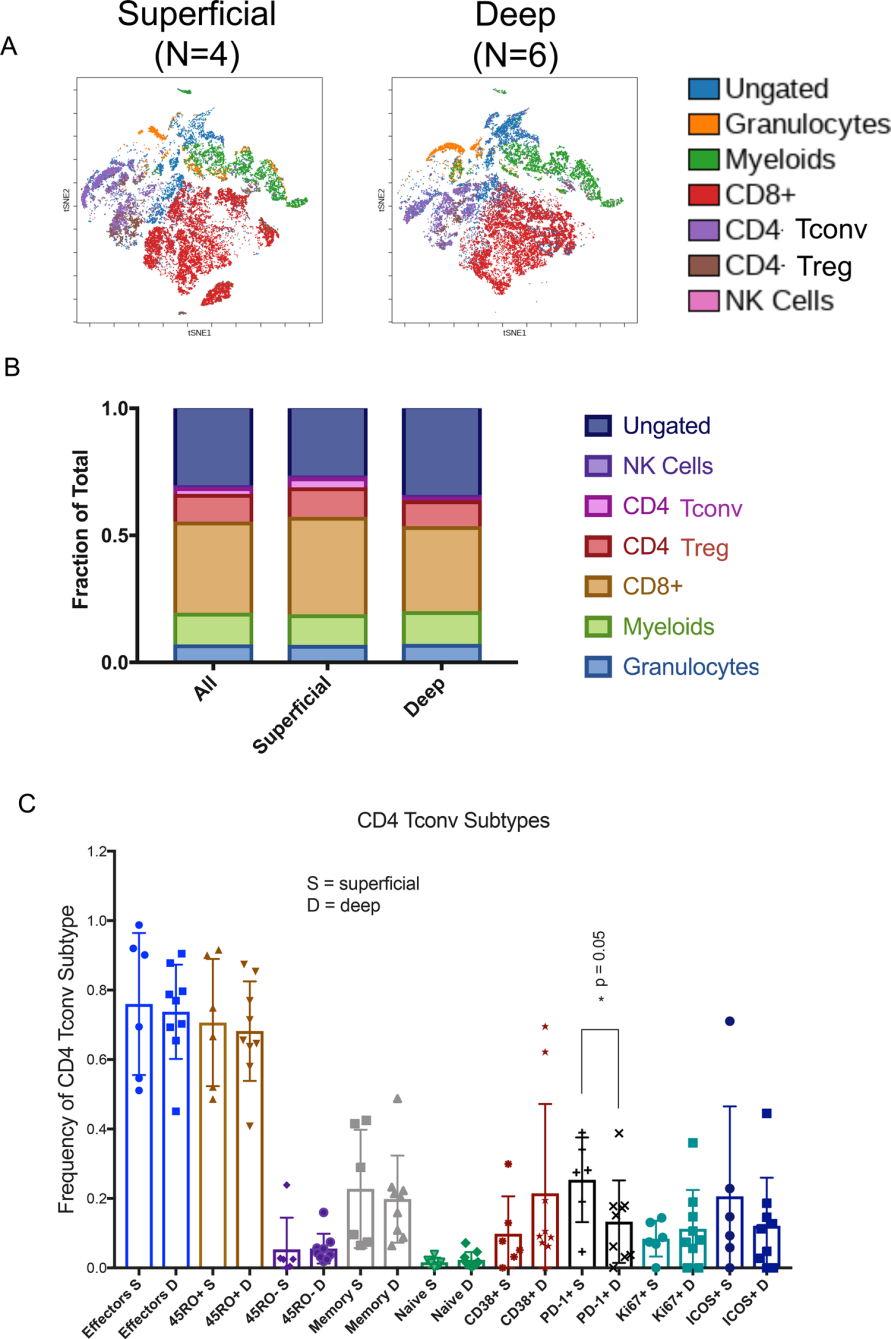
Impact of UPS tumor depth on tumor-infiltrating lymphocytes. **a** t-SNE plot analysis demonstrating immune cell subsets in superficial and deep UPS tumors. **b** Stacked bar graph demonstrating the fraction of immune-cell subsets in the entire UPS cohort, compared to superficial and deep UPS tumors. **c** Bar graph demonstrating the relative frequency of CD4 Tconv subtypes in superficial and deep UPS tumors. *p*-value, Mann–Whitney test

Beyond size, tumor depth is also an independent prognostic factor in UPS [4]. Similar to tumor size, our understanding of how tumor depth contributes to worse clinical outcomes remains undefined [22]. Evidence has shown that the anatomical compartment that tumors arise can provide a dynamic microenvironment that enables or suppresses tumor growth and progression [23, 24]. This is most evident in human sarcoma where tumors can arise in a spectrum of tissues, from subcutaneous to the deep muscle. Thus, we hypothesized that anatomical location of UPS influences tumor immune cell infiltration. To understand how the host immune response changes with respect to tumor depth, we stratified our UPS Cohort into tumors that arose in superficial (subcutaneous) or deep (subfascial) tissues. Through a comparative t-SNE analysis, we observed a modest decrease in the infiltration of total lymphocytes within the tumor microenvironment in deep seated UPS tumors compared to superficial tumors (Fig. 3a). We did not observe any relative decrease in other major immune cell subsets including TAMs or neutrophils (Fig. 3b). Further immunophenotypic classification revealed that a CD4 + T-cell population expressing PD1 decreased in deep seated UPS tumors compared to superficial tumors (Fig. 3c and Supplementary Fig. 1A–C). We have previously described the frequency of circulating CD4 + PD-1 + T cells associating with clinical response to immune checkpoint inhibition [25, 26]. Functionally, CD4 + FOXP3-PD-1 + (4PD1^hi^) TILs have been recently shown to limit effector T-cell functions, contributing to immune evasion [27]. The use of anti-PD-1 therapy counteracts the inhibitory function of 4PD1^hi^ cells and improves anti-tumor activity when used in combination with CTLA-4 blockade [27]. Thus, our findings demonstrate that deep and superficial UPS tumors may induce differential immune responses that can potentially guide future clinical stratification of UPS patients. Future immunoprofiling of the tumor microenvironment of UPS patients treated with anti-PD1 and anti-CTLA-4 therapies are needed to provide clinical insight into these biological observations.

## Discussion

Despite an improved understanding of the molecular events that promote UPS, the clinical prognosis of localized disease remains relatively unchanged for decades. In order to understand how conventional clinicopathologic features (tumor size and depth) are related to tumor-immune cell interactions in extremity UPS tumors, we performed single-cell immunoprofiling of the tumor-immune microenvironment of freshly resected, clinically annotated UPS tumors. Through this analysis, we reveal how physiologic (tumor size) and anatomical location (deep or superficial) shape the tumor-immune microenvironment and potentially impact the clinical outcome of UPS patients.

Our prospective analysis of UPS tumors revealed an inverse correlation between tumor size and effector CD8 + TIL abundance. Moreover, CD8 TIL abundance significantly correlated with improved overall survival in a retrospective cohort of UPS patients. These findings are consistent with previous data in non-mesenchymal tumors, where decreased CD8 + TILs is a negative prognostic marker for survival [6, 14–17, 28, 29]. Our findings propose a biological mechanism in that larger UPS tumors escape host immune surveillance, which is associated with fewer CD8 + TILs and potentially worse clinical outcomes in UPS patients. These data can potentially enhance the clinical stratification of UPS tumors, as the efficacy of checkpoint inhibitors depends on efficient effector T-cell infiltration into the tumor microenvironment [30]. Collectively, these data provide a descriptive framework to generate future hypotheses on how tumors mechanistically exclude CD8 + effector cells from the tumor-immune microenvironment in UPS tumors.

We observed a relative decrease in 4PD1^hi^ lymphocytes in UPS tumors located in deep anatomical compartments compared to superficial tumors. An immunosuppressive role for 4PD1^hi^ cells in the tumor microenvironment has recently been described [27]. Specifically, 4PD1^hi^ cells contribute to host immune evasion by limiting effector T cell functions [27]. Thus, these data suggest that deep and superficial UPS tumors may respond differently to immune-based therapies. We encourage future and ongoing clinical trials to stratify UPS patients based on superficial and deep-seated tumors to understand how the anatomical niche shapes the tumor microenvironment and potential response to checkpoint inhibitors.

Limitations of our study include the small sample size and short clinical follow-up of patients included in the prospective tissue acquisition cohort (average follow-up 20.2 months). Additionally the inclusion of a single patient treated with neoadjuvant radiation and chemotherapy can also be considered a limitation of this study. Notably, this patient represented a small fraction of our cohort (1 of 15); thus it does not influence the overall conclusions of our study. Notably, this patient’s tumor was resistant to radiation and chemotherapy as measured by tumor size (no radiographic response) and pathological necrosis (0%). Immunoprofiling of the resection specimen revealed a relatively low tumor infiltrating CD8 + lymphocyte population. These findings are consistent with prior studies that indicate that low CD8 + tumor lymphocyte infiltration following radiation exposure portends worse responses to radiotherapy [31]. Moreover, our retrospective analysis comparing CD8 + T-Cell expression in tumors from UPS patients either treated (N = 10) or not treated (N = 26) with neoadjuvant chemotherapy did not reveal a significant association (Supplementary Fig. 2).

Recent immune-based classification through transcriptional analysis (MCP-counter) of STS revealed an association between a B-Cell lineage signature and clinical outcomes in STS patients [32]. Through our multiparameter FACS-based approach, we did not observe an association between B-Cell markers (HLADR + /CD3-/CD19 +), tumor size, depth, or survival (Supplemental Fig. 3A–C). There are many potential explanations for these differences in B-cell associated outcomes observed in our population compared to the Petitprez dataset. First, we elected to focus on a single histological subtype (UPS). Thus, our findings cannot be directly compared to the heterogenous population (de-differentiated liposarcoma, Leiomyosarcoma, UPS) analyzed by Petitprez et al. Additionally, we used multiparameter FACS-based identification of immune-cell subsets in our prospective (freshly resected tumors) and targeted IHC analysis in our retrospective cohort. These approaches are distinct from the transcriptomic (MCP-counter tool) approach leveraged by Petitprez and colleagues.

Future studies will be aimed at identifying the intermediary factors that may influence tumor-immune cell profiles within deep and superficial tissues. These factors may include: (1) differential tissue resident immune cells and; (2) differences in native and tumor associated immune cell trafficking into the deep and superficial tissues. The establishment of a comprehensive atlas detailing the native host immune cell composition within normal (dermis, subcutaneous, and muscle) tissue compartments will potentially enable a deeper understanding of how UPS tumors influence the host immune response in humans. Thus, our findings provide an initial framework for future comparative studies that will enable future mechanistic pursuits to establish causative factors that directly influence UPS immune profile outcomes in different tissue compartments.

Through this analysis, we define how specific clinicopathologic parameters, including tumor size and depth shape the tumor-immune microenvironment in freshly resected UPS tumors. Future studies will be aimed at validating the clinical impact of these immune-based signatures in prospective trials.

## Materials and methods

### Prospective UPS cohort

Patients were enrolled onto two IRB approved biospecimen acquisition protocols at UCSF. The first (CC#16983) enabled prospective characterization of freshly resected UPS tumors. The second (CC#15981) provided investigators access to clinical information through a secure clinical database (REDCap). Patients were identified and consented to enroll on these studies if they had a biopsy-proven UPS scheduled for wide resection. An in-person translator and witness were employed for non-English speaking patients.

All sarcomas were resected with planned wide margins. The specimens were reviewed by an expert sarcoma pathologist, inked according to the marked orientation and sectioned to allow for analysis of margin status. The diagnosis of UPS was based on the WHO definition [33]. A 2 cm^3^ viable tumor section located away from the inked edge was obtained and sent for multiparameter flow cytometry (see below for specimen processing).

Clinical parameters from consented patients were entered into REDCap clinical database. These included patient age, gender, stage at diagnosis, pre-operative treatment including chemotherapy, radiation or prior surgery. Following resection, information from expert pathological review was entered including, percent necrosis, mitotic activity, tumor depth, and tumor size. The mean tumor size in the Discovery Cohort was 8 cm, which was used as a cut-off to stratify patients in this study. Follow-up clinical data was entered into the database at time of routine follow-up visits and included local recurrence, metastatic recurrence, adjuvant radiation or chemotherapy if used, death due to any cause and death due to disease.

### Single cell tissue processing

The resected tumor was minced using surgical scissors and then mechanically and enzymatically digested for 1 h at 37 °C using GentleMACS dissociation (Miltenyi Biotec), as per the TDK-1 tumor protocol provided by the manufacturer. The digestion reaction was then quenched using with DPBS + 2%FCS + 2 mM EDTA and filtered through a 100 um cell strainer. The cells were washed, resuspended in a 155 mM NH4Cl solution to lyse all erythrocytes, and washed.

### Flow cytometry

Cells were first incubated for 20 min at 4 °C with LIVE/DEAD™ Fixable Aqua Dead Cell Stain Kit (Invitrogen). After washing with DPBS + 2%FCS + 2 mM EDTA, samples were pre-incubated at 4 °C with Human TruStain FcX™ (BioLegend) and then stained with the respective antibody panel for 30 min at 4 °C. Cells were then washed and fixed either using BD Cytofix™ Fixation Buffer (Becton Dickinson) or eBioscience™ Intracellular Fixation & Permeabilization Buffer Set (Invitrogen) based on whether or not samples required subsequent intracellular staining. Stained cells were analyzed on a X-50 Fortessa flow cytometer (Becton Dickonson). Analysis files were first on FlowJo to exclude doublets, dead cells, and CD45- cells. The remaining CD45 + populations were exported as.fcs files and uploaded to Cytobank. Data was down-sampled for normalization and sequentially visualized using viSNE, a dimensionality reduction method based on the Barnes–Hut implementation of the t-SNE algorithm. The resulting plots were analyzed on Cytobank.

### Retrospective UPS cohort analysis

This retrospective study (CC#16871) was approved by the UCSF IRB. Clinically annotated patients diagnosed with UPS by an expert sarcoma pathologist between 2004 and 2012 were identified and included into this study. Thirty-six patients met inclusion criteria of minimum five-year follow-up and available tumor specimen. As with the prospective cohort, clinical characteristics including patient age, gender, stage at diagnosis, pre-operative treatment (chemotherapy, radiation or prior surgery), tumor size, percent necrosis, mitotic activity, tumor depth and clinical outcomes such as development of local recurrence, metastatic recurrence, adjuvant therapies received, death due to any cause and death due to disease. Overall survival (OS) was defined as time (in months) from the date of diagnosis to the time of death from any cause. OS was censored if the patient was still alive or lost to follow-up after minimum five-year follow-up period. Disease-free survival (DFS) was defined as time (in months) from the date of resection of the primary tumor to the date of either local or metastatic recurrence. DFS was censored if the patient did not develop local or metastatic recurrence or was lost to follow-up after minimum five-year follow-up period.

### Tissue microarray and archived UPS tissue specimens

Tumor specimens from nineteen patients in the UPS cohort were analyzed as a tissue microarray (TMA) developed at UCSF. The remaining seventeen patients had archived tumor specimens available as individual paraffin embedded tissue blocks or slides.

### IHC analysis

Histology was performed by HistoWiz Inc. (histowiz.com) using standard operating procedures and fully automated workflow. Briefly, paraffin embedded samples were sectioned at 4 μm. Immunohistochemistry was performed on a Bond Rx autostainer (Leica Biosystems) with enzyme treatment (1:1000) using standard protocols. Antibodies used were mouse monoclonal antihuman CD8 (Leica Biosystems, PA0183) and rabbit polyclonal CD20. Bond Polymer Refine Detection (Leica Biosystems) was used according to manufacturer’s protocol. Sections were then counterstained with Toluidine blue, dehydrated and film coverslip using a TissueTek-Prisma and Coverslipper (Sakura). Whole slide scanning (40x) was performed on an Aperio AT2 (Leica Biosystems).

### CD8 + T-cell and CD20 + B-cell quantification in Tissue.

The images were quantified using Halo image analysis software (Indica Labs) using CytoNuclear module to quantify CD8 and CD20 staining. All images were subsequently reviewed manually. For each TMA, all cores from UPS patients were selected as the annotated regions to be quantified, while for each slide, areas of high cellularity containing malignant cells were selected, avoiding fibrotic or pauci-cellular regions. The intensity for each stain was assigned under + 1, + 2, or + 3 category. Once set, the image analysis was completed on the annotated regions. For each annotated region, the image analysis software generated the following results: total cells, positive cells, negative cells, percentage of positive cells, percentage of negative cells, average stain one nuclear OD, average stain one cytoplasmic OD, average cell area, average cytoplasm area, average nucleus area, average nucleus perimeter, and tissue area (μm^2^). The percentage of positive cells was used as our primary measure of biomarker expression to allow for analysis of the cohort as a whole, including patients with specimens in the form of TMA and into survival analyses. For patients with TMA tumor specimens, the percentage of positive cells from triplicate cores was averaged.

### Statistical analyses

Univariate analysis using Cox regression was performed to identify clinical and pathologic factors that correlate with improved overall survival. Kaplan–Meier curves were used for survival analyses, and statistical significance of differences in survival due to differential expression of CD8 and CD20 was determined using the log-rank test. Assessment of the correlation between the number of CD8 + T lymphocytes and different clinicopathologic parameters was performed by using Spearman rank order correlation, Mann–Whitney U test, or Fisher-exact test as appropriate. All analyses were conducted by using SPSS version 24 software for Macintosh (SPSS, Chicago, IL). *P* less than 0.05 was identified as statistically significant.

### Supplementary Information

Below is the link to the electronic supplementary material.

Supplementary file1 (PDF 923 kb)
