# Supplementary material for: Tumor morphology and location associate with immune cell composition in pleomorphic sarcoma
Source: Cancer Immunol Immunother. 2021 Apr 17;70(10):3031–40. doi: 10.1007/s00262-021-02935-2 (PMC8423706; doi:10.1007/s00262-021-02935-2)
Supplement: Supplementary file 1 — Supplementary file1 (PDF 923 kb) [file 262_2021_2935_MOESM1_ESM.pdf]

Supplementary Material

Rosanna L. Wustrack, Evans Shao, Joey Sheridan, Melissa Zimel, Soo-Jin Cho, Andrew E. Horvai, Diamond Luong, Serena S. Kwek, Lawrence Fong, and Ross A. Okimoto

Tumor morphology and location associate with immune cell composition in pleomorphic sarcoma

Supplementary Material includes 3 figures and 5 tables.

Supplementary Figure 1

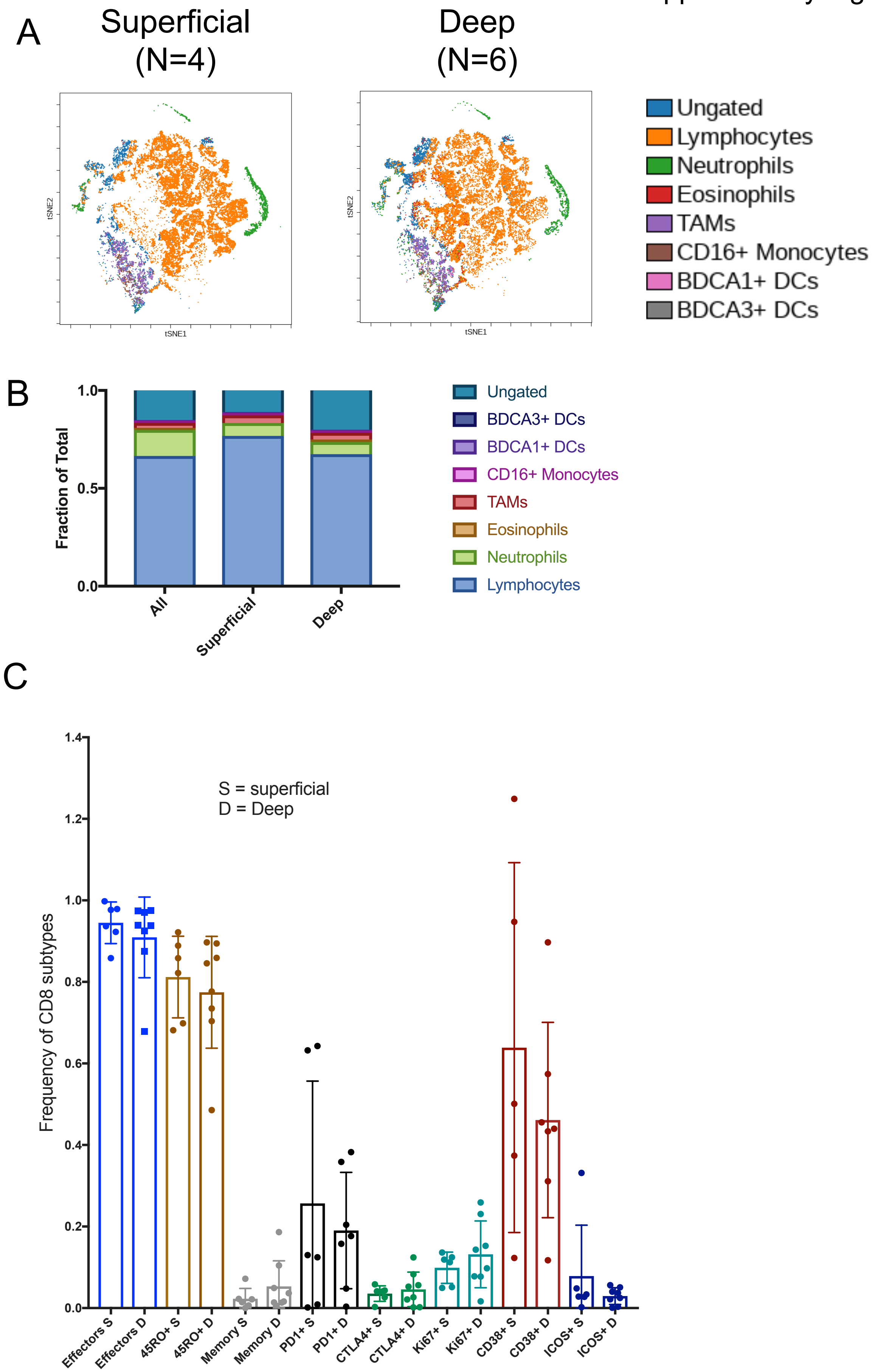

Supplemental Figure 1. Impact of UPS tumor depth on tumor infiltrating immune cell subsets.

A) t-SNE plot analysis demonstrating immune populations in superficial and deep UPS tumors. B) Stacked bar graph demonstrating the fraction of immune cell populations in the entire UPS cohort, compared to superficial and deep UPS tumors. C) Bar graph demonstrating the relative frequency of CD8 subtypes in superficial and deep UPS tumors.

Supplementary Figure 2

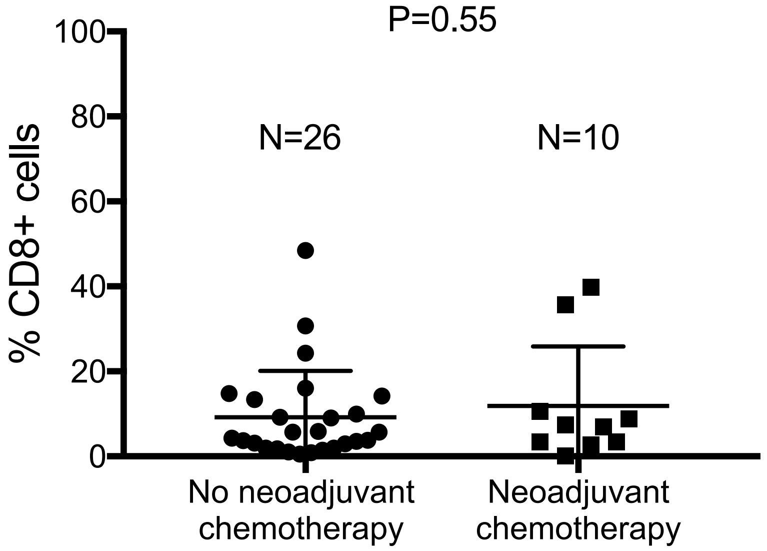

Supplementary Figure 2. % CD8+ T-Cells in UPS tumors derived from patients either treated or not treated with neoadjuvant chemotherapy.

Supplementary Figure 3

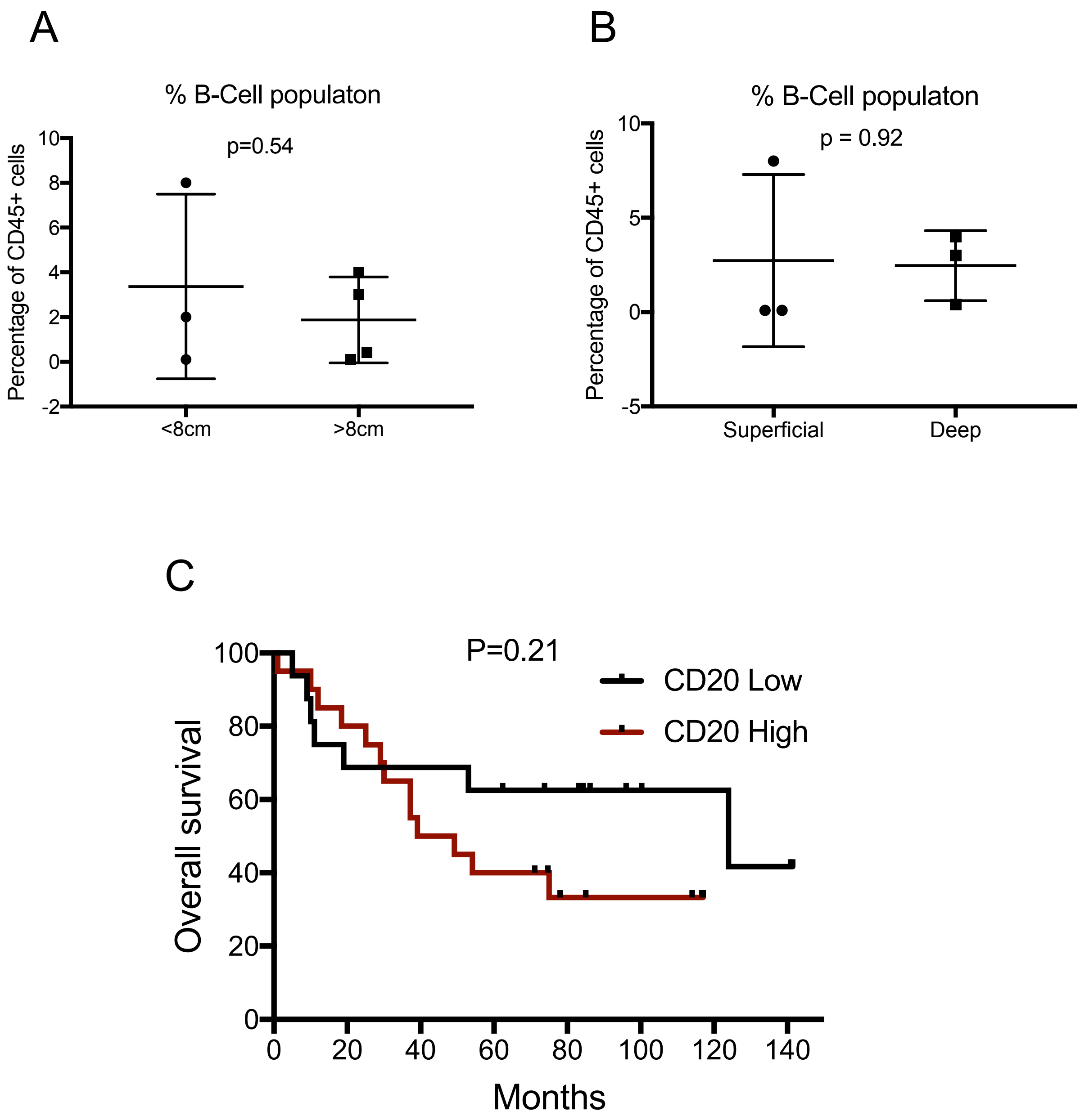

Supplementary Figure 3. B-Cell tumor infiltration and association with tumor size, depth, and survival.

% B-Cell (HLADR+/CD3-/CD19+) population in prospectively profiled UPS tumors < or > 8cm (A) and superficial or deep (B) location. C) Overall survival of UPS patients (retrospective cohort) with tumors expressing either high (N=20) or low (N=16) CD20 (B-Cell marker) positive B-Cells.

Supplementary Table 1. Demographics and neoadjuvant treatment N=15.

Patient	Gender	Age	Stage	Neoadjuvant Chemotherapy	Neoadjuvant radiation
C001	F	76	III	No	No
C004	F	84	II	No	No
C012	F	48	III	Yes	Yes
C014	M	67	IV	No	No
C016	M	70	III	No	No
C019	M	62	III	No	No
C022	F	57	II	No	No
C023	M	70	II	No	No
C026	M	76	III	No	No
C028	F	64	IV	No	No
C031	M	72	III	No	No
C034	M	85	IV	No	No
C036	F	66	III	No	No
C039	M	71	III	Yes	No
12519	M	75	II	No	No

Supplementary Table 2. Tumor Characteristics (N=15).

Patient	Tumor Size (cm)	Tumor Depth	Tumor Grade	Necrosis points 0-3	Mitotic Figures / 10 HPF
C001	5.3	Deep	3	1	22
C004	3	Superficial	3	0	35
C012	21.5	Deep	2	0	1
C014	9	Deep	3	1	50
C016	5.3	Deep	3	1	62
C019	34	Deep	3	1	41
C022	3	Superficial	3	1	33
C023	6.3	Superficial	3	1	21
C026	11.7	Deep	3	1	30
C028	8.8	Superficial	3	1	30
C031	6.9	Deep	3	1	22
C034	13.3	Deep	3	1	45
C036	6.5	Deep	3	0	40
C039	6.2	Deep	3	1	32
12519	6.5	Superficial	3	1	25

Supplementary Table 3. Clinical characteristics and follow-up (N=15).

Patient	FU (mo)	Adj. CTX	Adj. RT	LR	MR	Time to LR/MR (mo)	Death	Current Status
C001	43	No	Yes	No	Yes	8	No	AWD
C004	46	No	Yes	No	No		No	NED
C012	13	No	Yes	No	No		No	NED
C014	9	No	Yes	No	Yes	1	Yes	DOD
C016	31	No	Yes	No	No		No	NED
C019	14	No	Yes	Yes	No	27	Yes	DWD
C022	27	No	No	No	No		No	NED
C023	16	No	Yes	No	No		No	NED
C026	22	No	Yes	No	No		No	NED
C028	21	No	Yes	No	No		No	NED
C031	18	No	Yes	No	No		No	NED
C034	4	No	Yes	No	No		Yes	DOD
C036	14	No	Yes	No	No		No	NED
C039	15	Yes	No	No	Yes	4	No	AWD
12519	12	No	Yes	No	No		No	NED

FU = follow-up; mo = months; Adj. = adjuvant; CTX = chemotherapy; RT = radiation therapy; LR = local recurrence; MR = metastatic recurrence; AWD = alive with disease; NED = no evidence of disease; DOD = died of disease; DWD = died with disease.

Supplementary Table 4. Immune cell markers

Panel	Population Name	Corresponding Markers
T-cell	CD8 T Cell	CD45+/CD3e+/CD8+
T-cell	PD-1+ CD8	CD45+/CD3e+/CD8+/PD-1^{high}
T-cell	Ki67+ CD8	CD45+/CD3e+/CD8+/Ki67^{high}
T-cell	CTLA4+ CD8	CD45+/CD3e+/CD8+/CTLA4^{high}
T-cell	CD38+ CD8	CD45+/CD3e+/CD8+/CD38^{high}
T-cell	ICOS+ CD8	CD45+/CD3e+/CD8+/ICOS^{high}
T-cell	CD8 Effector	CD45+/CD3e+/CD8+/CD127-/CD25-
T-cell	CD8 Memory	CD45+/CD3e+/CD8+/CD127+/CD25-/CD45RO+
T-cell	CD8 Naïve	CD45+/CD3e+/CD8+/CD127+/CD25-/CD45RO-
T-cell	CD4 Thelper	CD45+/CD3e+/CD4+/CD25-/FoxP3-
T-cell	PD-1+ CD4 Thelper	CD45+/CD3e+/CD4+/CD25-/FoxP3-/PD-1+
T-cell	Ki67+ CD4 Thelper	CD45+/CD3e+/CD4+/CD25-/FoxP3-/Ki67+
T-cell	CTLA4+ CD4 Thelper	CD45+/CD3e+/CD4+/CD25-/FoxP3-/CTLA4+
T-cell	CD38+ CD4 Thelper	CD45+/CD3e+/CD4+/CD25-/FoxP3-/CD38+
T-cell	ICOS+ CD4 Thelper	CD45+/CD3e+/CD4+/CD25-/FoxP3-/ICOS+
T-cell	CD4 Effector	CD45+/CD3e+/CD4+/CD25-/FoxP3-/CD127-
T-cell	CD4 Memory	CD45+/CD3e+/CD4+/CD25-/FoxP3-/CD127-/CD45RO+
T-cell	CD4 Naïve	CD45+/CD3e+/CD4+/CD25-/FoxP3-/CD127-/CD45RO-
T-cell	T-regulatory	CD45+/CD3e+/CD4+/CD25+/FoxP3+
T-cell	NK Cells	CD45+/CD3e-/HLADR-/CD56+
T-cell	Myeloids	CD45+/CD3e-/HLADR+
Myeloid	Neutrophils	CD45low/SSC-A^{high}/CD16^{high}
Myeloid	Eosinophils	CD45low/SSC-A^{high}/CD16^{low}
Myeloid	Lymphocytes	CD45+/(CD56,CD19,CD20,CD3e) + (Dump Channel)
Myeloid	CD16+ Monocytes	CD45+/(CD56,CD19,CD20,CD3e)-/HLADR+/CD16+
Myeloid	Tumor Associated Macrophages (TAMs)	CD45+/(CD56,CD19,CD20,CD3e)-/HLADR+/CD16-/CD11c+/CD14+
Myeloid	Dendritic Cells (DCs)	CD45+/(CD56,CD19,CD20,CD3e)-/HLADR+/CD16-/CD14-/CD11c+
Myeloid	BDCA3+ DCs	CD45+/(CD56,CD19,CD20,CD3e)-/HLADR+/CD16-/CD14-/CD11c+/BDCA-3+
Myeloid	BDCA1+ DCs	CD45+/(CD56,CD19,CD20,CD3e)-/HLADR+/CD16-/CD14-/CD11c+/BDCA-1+

Supplementary Table 5. Neutrophil counts, lymphocyte Counts and Neutrophil-to-lymphocyte ratio (NLR).

	All (counts)	<8cm (counts)	>/=8cm (counts)
Neutrophils	12790	3524	9266
Lymphocytes	63880	35116	28764
NLR	0.20	0.10	0.32
